# Simultaneous bilateral intravitreal anti-vascular endothelial growth factor injections from one vial for diabetic macular edema: a retrospective analysis

**DOI:** 10.3389/fmed.2025.1712378

**Published:** 2025-10-23

**Authors:** Yuanmin Dai, Jixian Lou, Jiquan Wen, Guanlu Liang, Jiehui Xu, Zhiguo Jing, Juanjuan Wang

**Affiliations:** ^1^Department of Ophthalmology, Zhejiang Hospital, Hangzhou, Zhejiang, China; ^2^Huai’an Hospital of Huai’an City, Huai’an Clinical Medical College of Jiangsu University, Huai’an, Jiangsu, China

**Keywords:** intravitreal injection, diabetic macular edema, anti-vascular endothelial growth factors, safety, efficacy

## Abstract

**Objective:**

The objective of this study was to assess the safety and efficacy of simultaneous bilateral intravitreal injections of anti-vascular endothelial growth factors (anti-VEGF) from a single vial for the treatment of diabetic macular edema (DME).

**Methods:**

A retrospective case series study was undertaken. The study population comprised patients who underwent intravitreal anti-vascular endothelial growth factor injections for DME at the Ophthalmology department of Zhejiang Hospital between January 2022 and May 2024. Participants were categorized into bilateral (*n* = 34, 102 injections) and unilateral (*n* = 93, 210 injections) groups. The primary outcome measures were best corrected visual acuity (BCVA) and central subfield thickness (CST), cube average thickness (CAT) along with the incidence of severe clinical complications. Statistical analysis was performed using the Mann-Whitney U test and Wilcoxon rank sum test.

**Results:**

There were no statistically significant differences in terms of age, gender, hypertension, years of diabetes, disorganization of the inner retinal layers (DRIL), ellipsoid zone (EZ) grade, BCVA, CST or CAT at baseline between the two groups. Both bilateral and unilateral injection groups demonstrated significant enhancements in BCVA at 1 month post-injection (*P* < 0.001; *P* < 0.001). A substantial decrease in CST and CAT were observed in both groups at 1 month post-injection (*P* < 0.001; *P* < 0.001; *P* < 0.000; *P* < 0.000). The median improvement in BCVA at 1 month post-injection relative to pre-injection was 8.0 letters (IQR: 3.0–19.0) in the bilateral injection group and 5.5 letters (IQR: 1.0–13.0) in the unilateral injection group, with a statistically significant difference between the two groups (*P* = 0.009). The reduction in CAT demonstrated significant difference between the two groups (*P* < 0.001). No severe complications, including cataract, retinal detachment, choroidal detachment, vitreous hemorrhage, or endophthalmitis, were reported in any of the patients.

**Conclusion:**

Simultaneous bilateral anti-VEGF injections from a single vial are both safe and efficacious for the treatment of DME, thereby reducing the treatment burden, visit times and costs while enhancing patient compliance.

## 1 Introduction

The emergence of anti-vascular endothelial growth factor (Anti-VEGF) therapy has fundamentally altered the treatment paradigm for chorioretinal pathologies by targeting the aberrant angiogenesis and vascular permeability exacerbated by elevated VEGF levels. Currently, intravitreal Anti-VEGF injections represent the standard treatment modality for a range of conditions, including diabetic retinopathy (DR), diabetic macular edema (DME), age-related macular degeneration (AMD), retinal vein occlusion (RVO), and myopic choroidal neovascularization (mCNV) (1, 2). Intravitreal injection of anti-VEGF drugs has become the most commonly used treatment in ophthalmology, and its effectiveness and safety are confirmed (3–7). Many of these chronic diseases often occur in both eyes and have a long course, requiring multiple treatments and monitoring, the most common of which are AMD and DME (8, 9).

However, the transient therapeutic efficacy of these pharmaceutical agents necessitates their frequent and repetitive administration, thereby posing significant challenges to both patients and healthcare infrastructures (10). In clinical practice, the administration of a single dose to one eye is typically recommended to minimize the risk of complications, such as endophthalmitis. It is therefore not surprising that many patients express a strong preference for same-session bilateral injections over separate, unilateral injection sessions (11–13). Nonetheless, in resource-limited settings, such as those prevalent in developing nations, the implementation of this approach is often impeded by financial constraints, patient non-adherence, and limited access to medical services. The practice of conducting simultaneous bilateral injections from a single vial could overcome these challenges by reducing the number of visits required, cutting costs, and improving patient compliance with treatment regimens.

The purpose of this study is to investigate the safety and efficacy of conducting simultaneous bilateral intravitreal Anti-VEGF injections from a single vial in patients with DME, with the aim of providing empirical evidence to support its adoption into routine clinical practice.

## 2 Materials and methods

### 2.1 Patients source

This investigation constitutes a retrospective case series. Clinical data were meticulously compiled from patients who received intravitreal anti-vascular endothelial growth factor (anti-VEGF) injections at the Ophthalmology Center of Zhejiang Hospital from January 2022 to May 2024 for the treatment of Diabetic Macular Edema (DME). The study was conducted in accordance with the principles outlined in the Declaration of Helsinki, and all participants are exempted from the application of informed consent forms. Ethical approval was granted by the Ethics Committee of Zhejiang Hospital, under Approval No. 2024(093K).

Eligibility criteria encompassed individuals aged 18 years or older; those diagnosed with DME and subjected to intravitreal anti-VEGF therapy at our facility; and patients who had undergone comprehensive ophthalmic examinations with a follow-up period exceeding 4 weeks. Criteria for exclusion included cases managed for vitreous hemorrhage or neovascular glaucoma; instances where media opacity hindered OCT imaging, resulting in a signal strength below 6; incomplete or absent examination data; a follow-up period of less than 4 weeks; patients presenting with macular holes, retinal detachment, macular pseudoholes, age-related macular dystrophy, retinal vascular occlusion, intraocular inflammation and retinal dystrophies or other macular pathologies; and individuals with a history of ocular trauma or intraocular surgery during the follow-up interval. The cohort was subsequently segregated into two groups based on the injection modality: the unilateral injection group and the bilateral injection group.

The demographic data, clinical profiles, and medical histories of the patients were meticulously documented. Each participant underwent a comprehensive ophthalmological evaluation, encompassing the measurement of best-corrected visual acuity (BCVA) using Early Treatment Diabetic Retinopathy Study (ETDRS) charts, slit-lamp biomicroscopy, intraocular pressure (IOP) assessment, fundoscopy, and spectral domain-optical coherence tomography (SD-OCT) (Carl Zeiss Meditec Inc., CA). Central subfield thickness (CST) and cube average thickness (CAT) measurements were meticulously recorded. CST was quantified as the retinal thickness within the central 1-mm-diameter circle of the ETDRS grid. CAT was defined as the average thickness of the internal limiting membrane-retinal pigment epithelium tissue layer across the 6 mm× 6 mm square area that was scanned. SD-OCT-based grading systems were developed by prior research, wherein disorganization of inner retinal layers (DRIL) was characterized by the inability to distinguish the boundaries of the inner retinal layers and the ellipsoid zone (EZ) (14, 15).

### 2.2 Injection procedure

All interventions were executed by ophthalmologists possessing a minimum of 4 years of experience of clinical practice. The surgeons had completed standardized training specific to intravitreal injections. The intravitreal injections were administered in adherence to the hospital protocol, within the operating room (OR), under sterile conditions. Proxymetacaine hydrochloride 5% eye drops were instilled into the conjunctiva of each patient. The ocular region, inclusive of the eyelid skin and eyelashes, was sanitized with a 5% povidone-iodine solution 2 min prior to the procedure, followed by sterile draping. After donning surgical gowns and gloves, the anti-VEGF medication was aspirated into a 1 ml syringe at one time and subsequently the drug was extracted from the 1 ml syringe with an insulin syringe (29G Ultra-Fine BD insulin syringe) to 0.05 ml, which was divided into two 0.05 ml doses prior to patient contact. Each dose was positioned on the separated surgical field, with all instruments arranged accordingly. A sterile speculum was inserted to ensure the lashes were directed away from the eye. Proxymetacaine hydrochloride 5% drops were reapplied. Utilizing a caliper, the distance from the limbus was gauged: 3.5 mm for pseudophakic patients and 4 mm for phakic patients to administer the injection. For simultaneous bilateral injections, the identical preparatory measures were undertaken, and the surgeon then changed gloves and replicated the procedure. Subsequent to all injections, a single drop of topical antibiotic (levofloxacin) was administered onto the conjunctiva, and patients were directed to use post-injection topical levofloxacin eye drops four times daily for a duration of 5–7 days.

### 2.3 Data analysis

Statistical analyses were performed using IBM SPSS Statistics software (version 21.0). The normality of data distribution was determined via the Kolmogorov-Smirnov test. For categorical variables, percentages were presented; variables with non-normal distribution were described by the median, and variables with normal distribution were expressed as the mean and standard deviation. Normal distribution was tested by the chi-square test, and non-parametric methods were employed because of the non-normal distribution of the variables. Inter-group comparisons (bilateral injection group vs. unilateral injection group) were analyzed using the Mann-Whitney *U* test, while intra-group comparisons (pre-injection vs. post-injection) were evaluated with the Wilcoxon signed-rank test. A *P*-value less than 0.05 was regarded as statistically significant.

## 3 Results

**FIGURE 1:**
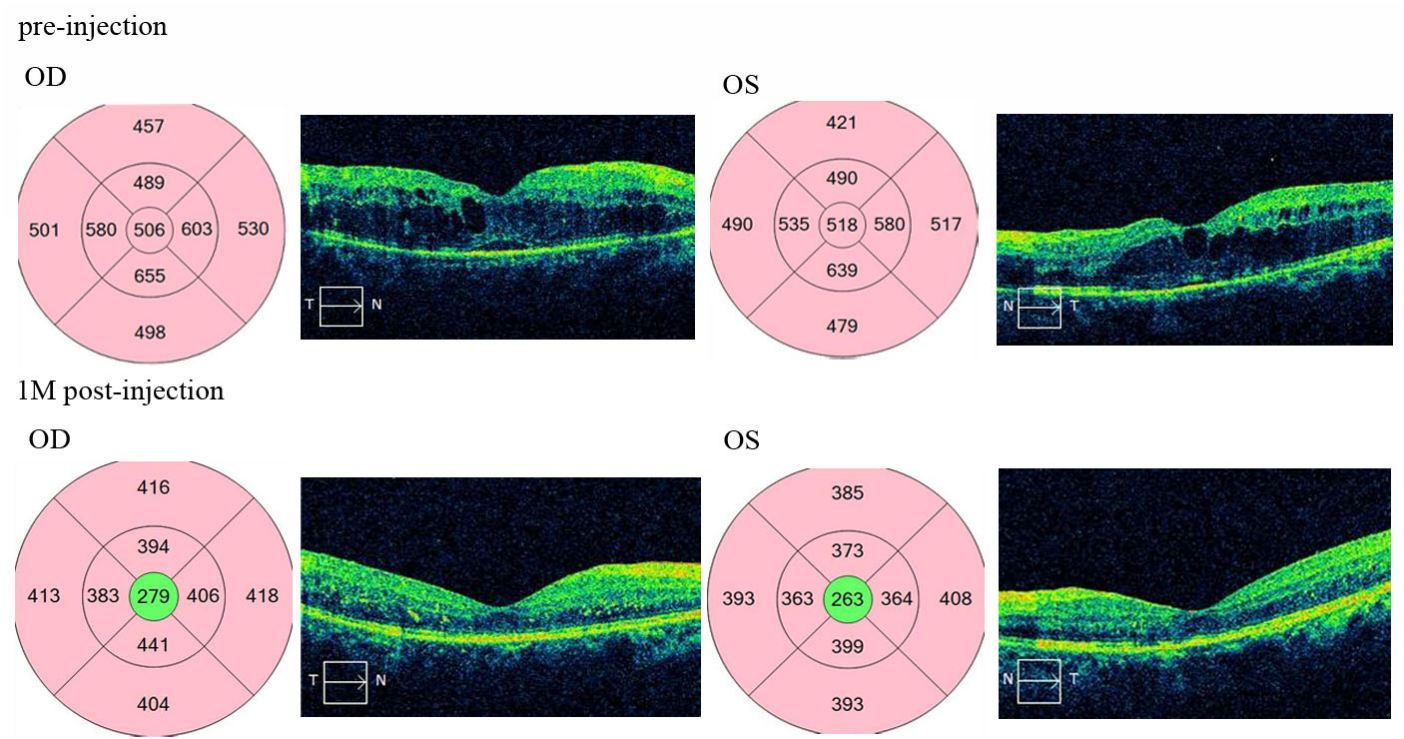
A 38-year-old male presented with blurred vision in both eyes for 1 month. He had a 5-year history of poorly controlled diabetes. Visual acuity was 47 letters in the right eye and 52 letters in the left eye. Macular OCT revealed bilateral diffuse edema and thickening in the macular region involving the fovea, with small amounts of subretinal fluid, disorganization of the retinal inner layers, cystoid edema, and focal disruption of the ellipsoid zone (EZ). One month after simultaneous bilateral intravitreal injections of anti-VEGF agents, visual acuity improved to 76 letters in the right eye and 80 letters in the left eye. Follow-up macular OCT showed resolution of macular edema in both eyes, disappearance of cystoid edema, absorption of subretinal fluid, and restoration of the retinal inner layer structure.

Finally, a total of 127 individuals received 312 injections, with the bilateral injection group comprising 34 individuals who received 102 injections, and the unilateral injection group comprising 93 individuals who received 210 injections. As detailed in Table 1, there were no statistically significant differences in terms of age, gender, hypertension, years of diabetes, disorganization of the inner retinal layers (DRIL), ellipsoid zone (EZ) grade, BCVA, CST or CAT before injection, when compared between the two groups. The median BCVA increased significantly from 56.0 letters (IQR: 43.5–61.0) before injection to 62.0 letters (IQR: 58.0–65.2) at 1 month (*P* < 0.001) in bilateral group, and increased significantly from 49.0 letters (IQR: 38.0–62.2) before injection to 61.5 letters (IQR: 49.0–68.0) at 1 month (*P* < 0.001) in unilateral group. The median CST decreased significantly from 352.0 μm (IQR: 278.7–460.0) before injection to 298.0 μm (IQR: 261.5–345.2) at 1 month (*P* < 0.001) in bilateral group, and decreased significantly from 313.5 μm (IQR: 273.7–418.0) before injection to 271.5 μm (IQR: 252.0–304.2) at 1 month (Table 2; *P* < 0.001) in unilateral group. The median CAT decreased significantly from 357.0 μm (IQR: 285.5–487.25) before injection to 298.0 μm (IQR: 256.7–342.0) at 1 month (*P* < 0.001) in bilateral group, and decreased significantly from 322.0 μm (IQR: 272.0–421.2) before injection to 270.0 μm (IQR: 251.0–304.3) at 1 month (Table 2; *P* < 0.000) in unilateral group (Figure 1). The median BCVA improvement and CAT reduction showed a statistically significant difference between the two groups, but CST reduction and showed no statistically significant difference between the two groups at the 1-month follow-up (Table 3; *P* = 0.009; *P* < 0.000; *P* = 0.081). No severe complications such as cataract, retinal detachment, choroidal detachment, vitreous hemorrhage, or endophthalmitis occurred in any of patients.

**TABLE 1:** Demographic, clinical and optical coherence tomography-based macular parameters data.

	Bilateral injection	Unilateral injection	*P*-value
Age (yrs), mean ± SD	50.54 ± 12.01	51.5 ± 11.59	0.6
Gender, (male/female)	18/16	54/39	0.687
Duration of diabetes (y), mean ± SD	10.82 ± 5.58	11.61 ± 6.26	0.842
Hypertension, *N* (%)	22 (64.7%)	56 (60.2%)	0.837
Pre-injection BCVA, median (Q1, Q3)	56.0 (43.5, 61.0)	49.0 (38.0, 62.2)	0.086
Pre-injection CST (μm), median (Q1, Q3)	352.0 (278.7, 460.0)	313.5 (273.7, 418.0)	0.087
Pre-injection CAT (μm), median (Q1, Q3)	357.0 (285.5, 487.25)	322.0 (272.0, 421.2)	0.464
DRIL, *N* (%)			0.975
Absent	14 (41.2%)	38 (40.8%)	
Present	20 (58.8%)	55 (59.2%)	
EZ grade, *N* (%)			0.186
Intact EZ	22 (64.7%)	72 (77.4%)	
Focal disruption	10 (29.4%)	15 (16.1%)	
Global disruption	2 (5.9%)	6 (6.5%)	
Aflibercept	34	63	
Conbercept	48	59	
Ranibizumab	20	88	

SD, standard deviation; N, number of patients; DRIL, disorganization of the inner retinal layers; EZ, ellipsoid zone. Variables with non-normal distribution were described by the median, and variables with normal distribution were expressed as the mean and standard deviation.

**TABLE 2:** Changes from pre-injection to 1M post-injection.

	Pre-injection median (Q1, Q3)	Post-injection median (Q1, Q3)	*P*-value
Bilateral injection BCVA (*n* = 102)	56.0 (43.5, 61.0)	62.0 (58.0, 65.2)	0.001**
Unilateral injection BCVA (*n* = 210)	49.0 (38.0, 62.2)	61.5 (49.0, 68.0)	0.001**
Bilateral injection CST (*n* = 102), (μm)	352.0 (278.7, 460.0)	298.0 (261.5, 345.2)	0.001**
Unilateral injection CST (*n* = 210), (μm)	313.5 (273.7, 418.0)	271.5 (252.0, 304.2)	0.001**
Bilateral injection CAT (*n* = 102), (μm)	357.0 (285.5, 487.25)	298.0 (256.7, 342.0)	0.000**
Unilateral injection CAT (*n* = 210), (μm)	322.0 (272.0, 421.2)	270.0 (251.0, 304.3)	0.000**

BCVA, best corrected visual acuity; CST, central subfield thickness; CAT, cube average thickness. Continuous data are presented as median (interquartile range).

**Values represent statistically significant alterations with *p* < 0.01 using Wilcoxon signed-rank test.

**TABLE 3:** Changes from pre-injection to 1M post-injection.

	Bilateral injection BCVA (*n* = 102) median (Q1, Q3)	Unilateral injection BCVA (*n* = 210) median (Q1, Q3)	*P*-value
BCVA improvement	8.0 (3.0,19.0)	5.5 (1.0,13.0)	0.009**
CST reduction, (μm)	47.5 (15.7,145.5)	33.0 (10.0,100.2)	0.081
CAT reduction, (μm)	54.5 (22.8,158.0)	37.0 (10.1,160.0)	0.001**

Continuous data are presented as median (interquartile range).

**Values represent statistically significant alterations with *p* < 0.01 using Wilcoxon signed-rank test.

## 4 Discussion

Anti-vascular endothelial growth factor (anti-VEGF) intravitreal injections have been employed for nearly two decades. These injections are characterized by their ease of administration, brief treatment duration, high standardization, well-defined clinical protocols, and controllable risks. To avert severe complications such as endophthalmitis, lens damage, and retinal detachment, it is recommended to perform single-eye, single-injection procedure. Nonetheless, in clinical practice, particularly for individuals with diabetic macular edema (DME), the necessity for multiple bilateral intravitreal injections presents significant challenges to patient adherence and imposes a considerable economic strain (16). To alleviate these issues, numerous clinicians opt to use a single vial to conduct simultaneous bilateral injections. However, diabetic patients, who often have compromised immune systems, are more susceptible to intraocular infections. This study aims to examine the safety and efficacy of conducting simultaneous bilateral intravitreal injections from a single vial within this specific patient population.

Due to the current economic situation and the need for treatment of patients, the demand for simultaneous injection of anti-VEGF drugs in both eyes is increasing. According to previous reports, intravitreal injection of anti-VEGF drugs is safe, but there are also reports of complications such as endophthalmitis (17). Kyuhwan et al. (18) undertook a retrospective analysis of 1,418 instances of simultaneous bilateral intravitreal injections administered to 646 eyes, revealing that all eyes exhibited enhancement in best-corrected visual acuity (BCVA) within 2 weeks post-injection, and no severe complications were documented (18). Audrey et al. (16) reported that patients with diabetic macular edema (DME) have a greater need for bilateral simultaneous injections both economically and socially, and reviewed that this treatment approach is safe and effective (16). According to previous reports, bilateral injections of anti-VEGF agents on the same day did not increase the rate of adverse events and increase visual outcome, was preferred by the majority of patients (19–21). Our research indicates that both bilateral and unilateral injections significantly improved BCVA and reduced CST and CAT, corroborating earlier academic studies. Although the reduction in CST did not attain statistical significance, a discernible trend toward a decrease was observed in the median values, which may be attributed to the limited sample size impacting the statistical power. Nevertheless, the cohort that received bilateral injections exhibited a more pronounced enhancement in BCVA and a greater reduction in CAT compared to the unilateral injection cohort. We posit that this occurrence may be akin to the “fellow-eye effect,” wherein small-molecule anti-VEGF drugs, known for their high permeability, achieve elevated intraocular drug concentrations in both eyes upon bilateral administration, leading to a concurrent diminution of macular edema and improvement in vision in both eyes (22–24). The simultaneous improvement in visual acuity in both eyes is postulated to yield a synergistic effect.

Previous research has documented an incidence of endophthalmitis ranging between 0.007 and 0.16% per injection, with the risk associated with simultaneous bilateral injections varying from 0.00 to 0.48% (25, 26). In previous reports, we found that endophthalmitis is a rare complication following bilateral same-session anti-VEGF injection therapy (27–32). Recent investigations conducted by Borkar et al. (33) and Grzybowski et al. (34) have substantiated the safety of same-day bilateral intravitreal anti-VEGF treatment, reporting no instances of endophthalmitis in a collective total of 1,612 bilateral injections. Jeeva et al. (30) documented a single occurrence of endophthalmitis (1/15,338) in the context of bilateral intravitreal anti-VEGF injections performed in an operating room, indicating no significant disparity in safety when compared to unilateral injections (30, 33–35). Se et al. (36) reported two cases of endophthalmitis in association with simultaneous bilateral injections utilizing a single vial; however, subsequent research from the same authors has indicated that this approach is safe when stringent aseptic principles are observed during the injection procedure (36). Studies have shown that simultaneous intravitreal injection of anti-VEGF drugs in both eyes is safe in various types of diseases (37–39), even if the drug is dispensed from a single bottle. In our study, no severe complications such as cataract, retinal detachment, choroidal detachment, vitreous hemorrhage, or endophthalmitis were noted during the follow-up period, aligning with the findings of prior literature. Consequently, simultaneous bilateral intravitreal injections employing a single vial are deemed safe within an operating room environment, provided that aseptic techniques are rigorously maintained and each eye is injected independently. This holds true even for diabetic patients. Patients experienced good tolerance, as bilateral injections reduce treatment expenses, enhance visual acuity in both eyes concurrently, diminish the frequency of follow-up appointments and injections, and thereby increase patient satisfaction.

It is imperative to acknowledge the inherent limitations of this investigation. Specifically, it is a retrospective case series that does not possess a sufficient number of injections to precisely reflect the incidence of endophthalmitis. The complications arising from intravitreal injections, including subconjunctival hemorrhage, uveitis, transient intraocular pressure elevation, and allergic reactions, were not subjected to comprehensive comparison within the scope of this study. Additionally, the indicators employed for detection were not all-encompassing. Future research endeavors should be designed as prospective studies and should include a more substantial cohort to further elucidate the safety and efficacy profiles of simultaneous bilateral intravitreal anti-VEGF injections derived from a single vial.

## 5 Conclusion

In summation, the present study conducted a retrospective assessment of the safety and efficacy associated with the administration of simultaneous bilateral intravitreal anti-vascular endothelial growth factor (anti-VEGF) injections from a single vial for the management of diabetic macular edema (DME). The findings suggest that this approach is both safe and efficacious, leading to a decrease in the financial strain on individuals with diabetes, a reduction in the frequency of follow-up appointments, and a diminution in overall healthcare expenditures. The synergistic effect of improving vision in both eyes may lead to better visual benefits.

## Data Availability

The datasets presented in this study can be found in online repositories. The names of the repository/repositories and accession number(s) can be found in this article/supplementary material.
